# Bioinformatics Analysis of Biomarkers and Therapeutic Targets Related to Necroptosis in Intervertebral Disc Degeneration

**DOI:** 10.1155/bmri/9922966

**Published:** 2024-12-16

**Authors:** Fan Zhang, Lei Yuan, Heng Ding, Zhenkai Lou, Xingguo Li

**Affiliations:** Department of Orthopedics, The First Affiliated Hospital of Kunming Medical University, Kunming City, Yunnan 650032, China

**Keywords:** ceRNA network, diagnostic markers, immune infiltration, intervertebral disc degeneration, necroptosis, therapeutic target

## Abstract

Necroptosis is a critical process in intervertebral disc degeneration (IDD). This research is aimed at identifying key genes regulating necroptosis in IDD to provide a theoretical basis for early diagnosis and treatment. Transcriptome data from patients with IDD and normal samples were obtained from the GSE34095 and GSE124272 datasets of the Gene Expression Omnibus (GEO) public database. Necroptosis-related genes (NRGs) were sourced from the GeneCards database and literature. Differentially expressed necroptosis-related genes (DE-NRGs) in IDD were identified by intersecting these sources. Gene Ontology (GO) and Kyoto Encyclopedia of Genes and Genomes (KEGG) were used for gene annotation analysis. The receiver operating characteristic (ROC) curve and nomogram analyses assessed the diagnostic efficiency of DE-NRGs. The miRWalk and starBase databases helped construct the competing endogenous RNA (ceRNA) regulatory network of DE-NRGs. We identified 517 differential genes in tissue and 2974 in blood, with 62 genes in common. DE-NRGs (*AIFM1*, *CCT8*, *HNRNPA1*, *KHDRBS1*, *SERBP1*) were identified by intersecting NRGs with these 62 common genes. The ROC curve showed an area under the curve (AUC) > 0.70 for DE-NRGs, and the nomogram indicated that a higher DE-NRG score correlates with a higher risk of IDD. *CCT8*, *KHDRBS1*, and *AIFM1* emerged as potential therapeutic targets for IDD through target drug prediction. qRT-PCR (quantitative reverse transcription polymerase chain reaction), Western blot, and immunohistochemistry confirmed the expression of *AIFM1*, *CCT8*, *HNRNPA1*, *KHDRBS1*, and *SERBP1* in patients' nucleus pulposus tissue, suggesting these genes as key targets for IDD risk assessment and drug therapy.

## 1. Introduction

Intervertebral disc degeneration (IDD) is the pathological basis of spinal degenerative disease, which is caused by many factors, including biomechanical factors, nutritional supply disorders, and genetics. As the degree of degeneration deepens, diseases such as herniation of the intervertebral disc (IVD), stenosis of the spinal canal, and spondylolisthesis will occur in the spine, resulting in low back pain. At present, the clinical diagnosis and treatment methods and the therapeutic effects are very limited; especially for early IDD, there is still a lack of effective intervention methods. The IVD consists of a central elastic gelatinous nucleus pulposus (NP), an annulus fibrosus (AF) in the surrounding region, and upper and lower cartilaginous endplates (CEPs) [1]. Increasing research indicates that cell death within the IVD, especially NP cell death, plays a crucial role in IDD pathogenesis [2]. While apoptosis has been considered the primary mode of NP cell death, therapeutic strategies targeting apoptosis inhibition have yielded unsatisfactory results, suggesting the involvement of other cell death mechanisms. Necroptosis, a novel form of regulated cell death, is closely linked to apoptosis [3]. In IDD, if apoptosis of myeloid cells in the IVD is inhibited, the apoptotic pathway may progress to necroptosis [4]. Therefore, further exploration of the mechanisms underlying necroptosis and IDD is essential for developing effective IDD treatments.

Necroptosis is a form of programmed cell death triggered by severe cytotoxic damage, characterized by cell swelling, loss of membrane integrity, DNA degradation, and release of cytoplasmic contents [5]. This process is cysteine dependent and requires the kinase activity of receptor-interacting protein kinase 1 (RIPK1) and receptor-interacting protein kinase 3 (RIPK3) [6]. Necroptosis is a form of programmed cell death initiated by severe cytotoxic stress, characterized by cellular swelling, loss of membrane integrity, DNA fragmentation, and the release of cytoplasmic contents. This process is dependent on cysteine and necessitates the kinase activities of RIPK1 and RIPK3. Mixed lineage kinase domain-like (MLKL) pseudokinases serve as downstream targets of RIPK3 and are the ultimate effectors in the execution of necroptosis. Upon activation, RIPK3 phosphorylates MLKL, inducing a conformational change in the pseudokinase domain of MLKL. This modification facilitates the translocation of MLKL from the cytoplasmic lysosome to various cellular membranes, culminating in membrane disruption and subsequent necrosis [7]. Over the past decade, necroptosis has been implicated in various diseases, including ischemic injury, neurodegenerative disorders, and acute chest syndromes. Increasing evidence suggests that necroptosis plays a significant role in IDD [6]. For instance, stress can induce necroptosis in NP-derived stem cells, hindering the endogenous repair of IDD [8]. Hydrostatic pressure may lead to mitochondrial dysfunction and oxidative stress by upregulating RIPK expression, promoting NP cell necrosis, and consequently inducing IDD [9]. The PARK2 gene has been identified as potentially influencing IDD progression through methylation modifications, although research on genes related to cell necrosis remains limited [10]. If cell death is a causative factor in degenerative diseases, reversing the corresponding pattern could offer therapeutic benefits. Therefore, further investigation into the molecular mechanisms of IDD-associated necroptosis is crucial for developing prevention and intervention strategies.

In this study, we screened key genes related to necrotic apoptosis in IDD using public transcriptome databases. We utilized online databases miRWalk and starBase for competing endogenous RNA (ceRNA) relationship pair prediction, constructing both the ceRNA regulatory network and the regulatory network of key genes. Potential therapeutic drugs were predicted using CLUE COMMAND. This research is aimed at providing a theoretical basis for identifying potential therapeutic targets for IDD.

## 2. Materials and Methods

### 2.1. Data Sources

We utilized the Gene Expression Omnibus (GEO) database (https://http://www.ncbi.nlm.nih.gov/geo/) to obtain single-cell sequencing data for IDD from datasets GSE34095 and GSE124272. GSE34095 includes 6 samples (IDD = 3, control = 3), while GSE124272 involves 16 blood samples (IDD = 8, control = 8). Initially, we performed Spearman correlation analysis on the two datasets separately and selected pairs with correlations present in both datasets for further analysis.

The miRNAs of target key genes (GSE116726) were predicted by the miRWalk database. The targeting relationship between lncRNA and the miRNA (clipExpNum > 1, geneType = lincRNA) was predicted by the starBase database (https://starbase.sysu.edu.cn/index.php). The online database NetworkAnalyst (https://www.networkanalyst.ca/) was used to predict the regulatory factors of key genes.

### 2.2. Patient Samples

This study was approved by the Ethics Committee of the First Affiliated Hospital of Kunming Medical University (No. (2023) Lun Shen L No. 176), in accordance with the Declaration of Helsinki, and informed consent was obtained from all patients. We collected NP specimens from 12 patients with IDD, comprising 7 males and 5 females, with an average age of 50.14 ± 13.36 years. Three senior surgeons classified the patients into mild IDD (M-IDD) (Grade II/III) and severe IDD (S-IDD) (Grade IV/V) based on the improved Pfirrmann grading system and magnetic resonance imaging (MRI) [11]. We validated our results using qRT-PCR (quantitative reverse transcription polymerase chain reaction) and immunohistochemistry (IHC) experiments.

### 2.3. Differential Gene Expression and Functional Analysis

The datasets GSE34095 and GSE124272 were used to screen differential gene expressions of IDD and control (blood and IVD tissue) (*p* < 0.05), based on the R language limma package (Version 3.46.0). The differentially expressed genes (DEGs) were obtained from the volcano map by using package ggplot2 (Version 3.3.4). The heat map of top 50 genes was drawn for visualization by using pheatmap (Version 1.0.12). The clusterProfiler package (Version 3.18.0) was used for the functional enrichment analysis of common differential genes (*p* < 0.05, count ≥ 1), and the enrichPlot (Version 1.10.2) was used to make a bar chart or bubble chart. Moreover, ClueGO was used to analyze the relationship between protein functions.

#### 2.3.1. Screening of Differentially Expressed Necroptosis-Related Genes (DE-NRGs)

From the GeneCards database (https://www.genecards.org/) and the reported literatures [12, 13], we obtained necroptosis-related genes (NRGs). And then, the genes screened from the database and literatures were intersected to achieve the DE-NRGs.

### 2.4. Evaluate the Diagnostic Efficiency of DE-NRGs for IDD

The single gene receiver operating characteristic (ROC) curve was analyzed by pROC (Version 1.17.0.1). As a whole, the key genes were used to build a logical regression (LR) model, random forest (RF) model, support vector machine (SVM) model, and artificial neural network (ANN) model to estimate the diagnostic efficiency of overall DE-NRGs for IDD. Each DE-NRG was scored on the nomogram to predict the risk ratio of experiencing IDD. The rmda package was used to draw the decision curve analysis (DCA) of DE-NRGs, to intuitively evaluate the clinical effect of the nomogram model.

### 2.5. Construction of ceRNA Network

miRWalk is used to predict miRNA targeting of key genes, and starBase is used to predict the targeting relationship between miRNAs and lncRNAs. The miRNA, lncRNA, differentially expressed miRNA (DE-miRNA), and differentially expressed lncRNA (DE-lncRNA) predicted in the database were intersected to obtain the target relationship pairs. Finally, the ceRNA network was visualized by Cytoscape.

### 2.6. Therapeutic Drug Prediction Methods

The online database CLUE COMMAND (https://clue.io/) is used to predict the targeted drugs related to key genes which the small molecule compounds with potential therapeutic effects in IDD cases.

### 2.7. Patient Sample Validation

#### 2.7.1. qRT-PCR Validation

Total RNA was extracted from NP using the RNA extraction and separation kit (Thermo Fisher), and mRNA first-strand cDNA (Takara) was synthesized using the same kit. Double-stranded DNA was amplified using the qPCR kit (Takara), and GAPDH was used as the control *ΔΔ* CT (cycle threshold) analysis. All primers were synthesized by Tsingke Biotechnology (Beijing, China) (Table 1). qRT-PCR was used to detect the expression levels of *AIFM1*, *CCT8*, heterogeneous nuclear ribonucleoprotein A1 (*HNRNPA1*), signal transduction-associated 1 (*KHDRBS1*), and sterol regulatory element–binding protein 1 (*SERBP1*) genes in the NP.

Western blot was used to detect the phosphorylation level of MLKL in Ser358 (ab187091), and IHC was used to detect the expression level of apoptosis-related gene MLKL in NP tissue, in order to comprehensively evaluate the level of necrotic apoptosis in NP cells.

### 2.8. Statistical Analysis

The data were analyzed using R and GraphPad Prism software. Each value corresponds to data from three independent experiments and is expressed as mean ± standard deviation. Comparisons between multiple groups were analyzed using one-way ANOVA. A *p* value of less than 0.05 was considered statistically significant.

## 3. Results

### 3.1. Differential Gene Screening and Function Prediction

**Figure 1:**
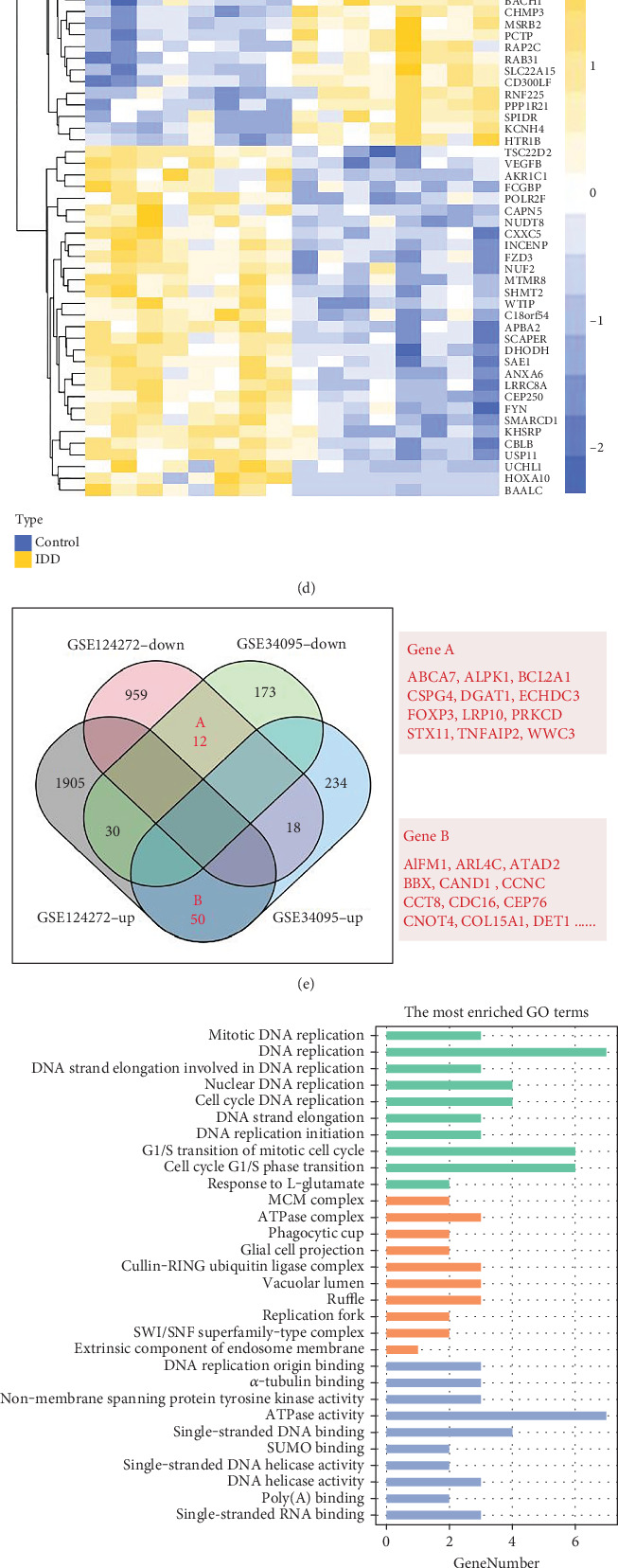
The screen and function analysis of common DEGs. (a) The volcano map of DEGs in GSE34095. (b) The heat map of GSE34095 DEGs. (c) The volcano map of GSE124272 DEGs. (d) The heat map of GSE124272 DEGs. (e) The Venn analysis of GSE34095 and GSE124272 DEGs intersecting. (f) The GO enrichment results of common DEGs. (g) The GO functional connection of common DEGs. (h) The KEGG enrichment term of common DEGs. (i) The PPI interactivity network of common DEGs. Logistic regression (LR), random forest (RF), support vector machine (SVM).

Based on information from the two databases, we screened for DEGs. Through differential analysis of tissue samples, we identified a total of 517 DEGs (DEG1), with 302 genes upregulated and 215 genes downregulated in IDD (Figures 1(a) and 1(b)). In blood samples, we found 2974 DEGs (DEG2), with 1985 genes upregulated and 989 genes downregulated in IDD samples (Figures 1(c) and 1(d)). Due to the heterogeneity of IDD tissue and blood samples, we conducted interaction analysis to ensure that the genes differed in both tissues and blood, thereby reducing the impact of heterogeneity. This analysis revealed a total of 62 genes with significant changes in both blood and tissue samples, comprising 12 downregulated and 50 upregulated genes (Figure 1(e)).

We further performed biological enrichment analysis on these DEGs. The cell biological process (BP) analysis indicated that these genes were significantly related to the G1/S phase transition of the cell cycle, the G1/S phase transition of the mitotic cell cycle, and the response to L-glutamate. In terms of cell composition (CC), the common differential genes were significantly associated with exogenous components of membranes in vivo, SWI/SNF superfamily-type complexes, and replication forks. Regarding molecular function (MF), they were significantly related to single-stranded RNA binding, poly(A)-binding, and DNA helicase activity (Figures 1(f) and 1(g)). Additionally, the Kyoto Encyclopedia of Genes and Genomes (KEGG) analysis revealed that these genes were mainly involved in ubiquitin-mediated protein hydrolysis, cell cycle, DNA replication, glutathione metabolism, drug metabolism–other enzymes, and nucleotide metabolism (Figure 1(h)). To explore interactions between shared differential genes, we constructed a protein–protein interaction (PPI) network for the 62 shared genes. The PPI network interactions showed that 36 differential genes interacted with each other, with MCM6, MCM4, and RRM1 at the core of the PPI graph (Figure 1(i)), indicating a relatively high degree of interactions. These results suggest that the differential genes in IDD are primarily related to the cell cycle and may influence the development of IDD by regulating cell survival and death.

### 3.2. Acquisition and Functional Analysis of Key Genes of Necroptosis

**Figure 2:**
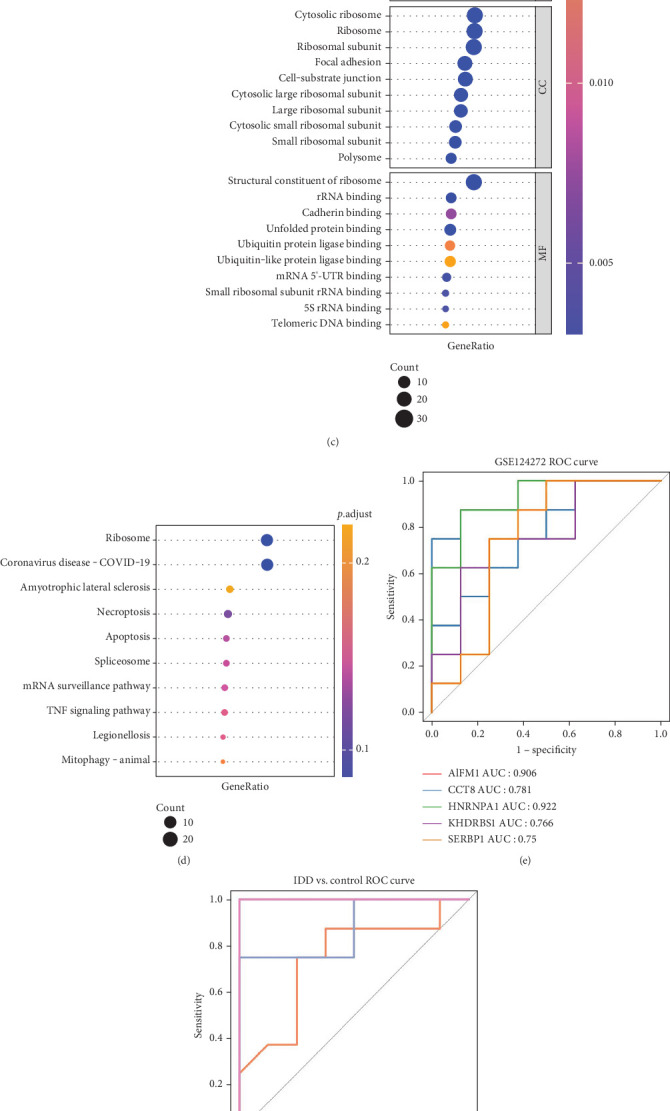
The screen function analysis and evaluation of DE-NRGs. (a) The Venn map. (b) The coexpression network of DE-NRGs with others NRGs. (c) The GO enrichment bubble map of DE-NRGs and coexpression genes NRGs. (d) KEGG enrichment bubble map of DE-NRGs and coexpression NRGs. (e) The diagnosis ROC curve of DE-NRGs. (f) The ROC curve of multiple diagnostic models. (g) Nomogram of DE-NRGs. (h) The DCA curve to evaluate the clinical efficiency of the nomogram model. (i) The clinical influence curve of nomogram.

As shown in the functional section above, the shared differential genes were enriched to some apoptosis and cell cycle–related pathways; next, we focused on apoptosis and analyzed necrotic apoptosis. We obtained a total 643 NRGs from different literatures and databases. To obtain DE-NRGs, we took the intersection of 643 NRGs with 62 shared DEGs. We obtained a total of five DE-NRGs by analyzing them, which were *AIFM1*, *CCT8*, *HNRNPA1*, *KHDRBS1*, and *SERBP1* (Figure 2(a)). Additionally, we explored the correlation of these five DE-NRGs with other necrotic genes using Spearman correlation analysis. The results showed that the five DE-NRGs were moderately correlated with other NRGs, with *KHDRBS1* exhibiting the strongest correlation (Figure 2(b)). Our functional enrichment analysis of the genes in the network from Figure 2(a) revealed significant associations with BPs such as endoplasmic reticulum protein localization, viral gene expression, viral transcription, and translation initiation. In terms of MF, they were significantly associated with telomeric DNA binding, 5S rRNA binding, mRNA 5⁣′-UTR binding, and ubiquitin-like protein ligase binding (Figure 2(c)). Meanwhile, KEGG analysis indicated that these genes were mainly associated with mitosis, the TNF signaling pathway, mRNA regulatory pathways, spliceosome, apoptosis, necrosis, ribosomes, and more (Figure 2(d)).

To assess the diagnostic value of DE-NRGs for IDD patients, we plotted ROC curves and calculated area under the curve (AUC) values. The analysis results showed that the AUC values of five DE-NRGs were greater than 0.7 (Figure 2(e)), and the AUC values of LR, RF, SVM, and ANN models were greater than 0.7 (Figure 2(f)). This suggests that DE-NRGs have good diagnostic potential for IDD. In addition, we used logistic regression to construct a nomogram for the five key genes identified, facilitating clinical assessment of IDD risk (Figure 2(g)). In the nomogram, a higher score indicates a higher risk of IDD.

### 3.3. ceRNA Regulatory Network and Transcription Factor (TF) Regulatory Network of Key Genes

**Figure 3:**
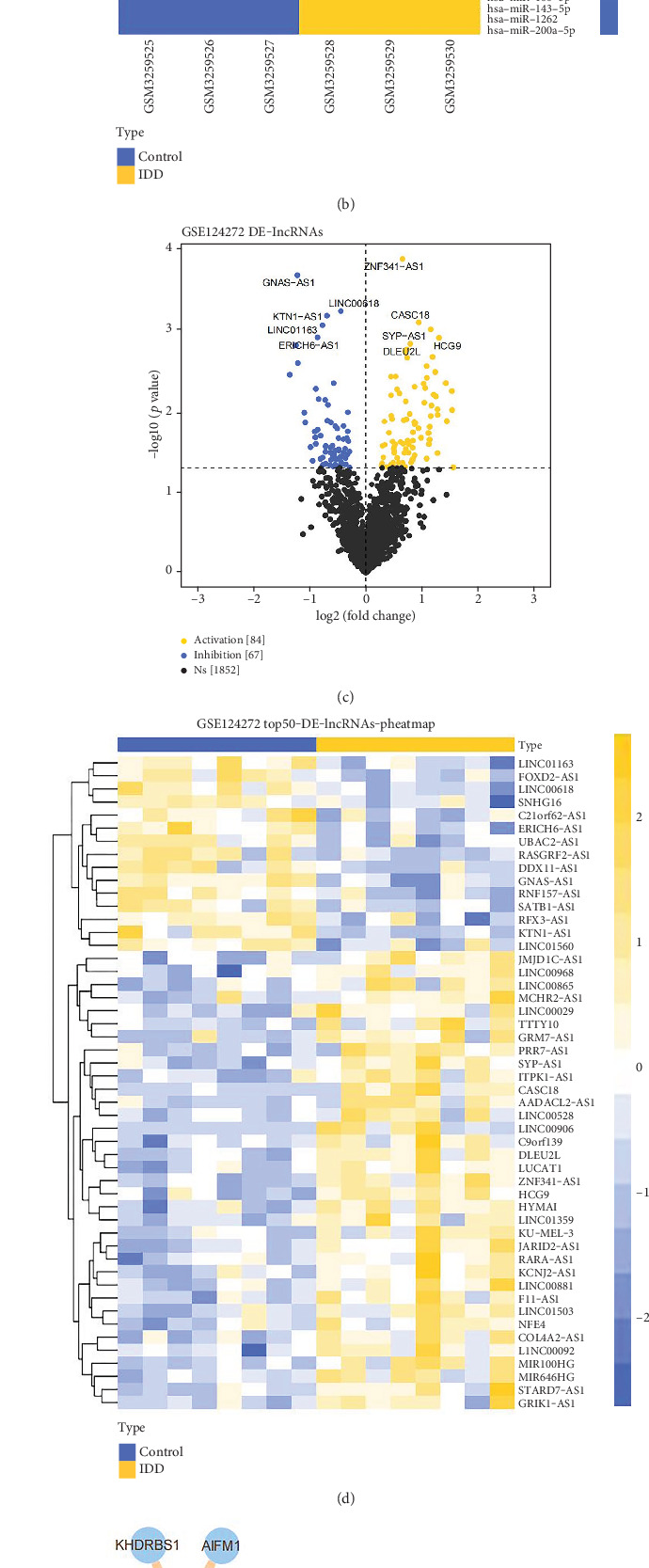
ceRNA regulated network where construct and treatment drugs were predicted of DEGs. (a) Volcano map of DE-miRNA in GSE116726. (b) Heat map of DE-miRNA in GSE116726. (c) Volcano map of DE-miRNA in GSE124272. (d) Heat map of DE-miRNA in GSE124272. (e) ceRNA regulated network of DE-NRGs. (f) A volcano map of DE-IFs. (g) A regulated network of DE-NRGs-IF.

It is well known that microRNAs can cause gene silencing by binding to mRNAs, while ceRNAs can regulate gene expression by competitively binding to microRNAs. To explore the potential regulatory targets of key genes, we predicted miRNAs targeting key genes using the miRWalk database and differentially analyzed miRNAs against the GSE116726 and GSE124272 databases. Through the analysis, we obtained 508 DE-miRNAs (Figures 3(a) and 3(b)) and 151 DE-lncRNAs (Figures 3(c) and 3(d)). Then, we took the intersection of predicted miRNAs with DE-miRNAs; since DE-NRGs were upregulated, the relationship pairs between lncRNA–miRNA–mRNA were investigated by preserving the downregulated miRNA and the upregulated lncRNA (Table 2). The results showed that the lncRNA–miRNA–mRNA network contained 4 key genes, 11 miRNAs, and 2 lncRNAs (Figure 3(e)). The ceRNA results suggested that the above miRNAs and lncRNAs were important targets for regulating the expression of key genes.

Moreover, we also predicted the regulators of the key genes and obtained 36 different TFs by crossing the predicted TFs and DEGs (GSE124272) (Figure 3(f)). We further constructed a regulatory network of these 36 TFs with the key genes (Figure 3(g)). Overall, the results suggest that the analyzed miRNAs, lncRNAs, and TFs may be significant targets for regulating the expression of key genes in IDD.

### 3.4. Potential Therapeutic Drug Prediction

**Figure 4:**
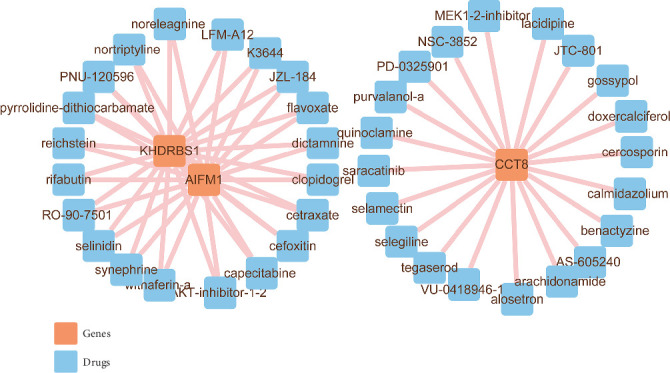
The key gene–treatment drug network in database CLUE COMMAND.

To identify potential therapeutic agents for IDD, we predicted target drugs for five key genes using the CLUE COMMAND database. The analysis revealed that CCT8 showed strong correlations with drugs such as selamectin, cercosporin, calmidazolium, doxercalciferol, benactyzine, and PD-0325901. Additionally, *KHDRS1* and *AIFM1* were strongly correlated with drugs like capecitabine, flavoxate, cefoxitin, cetroxate, dictamine, clopidogrel bisulfate, and AKT inhibitor 1–2, among others (Figure 4). The effects of these drugs on IDD warrant further exploration.

### 3.5. Patient Sample Validation

**Figure 5:**
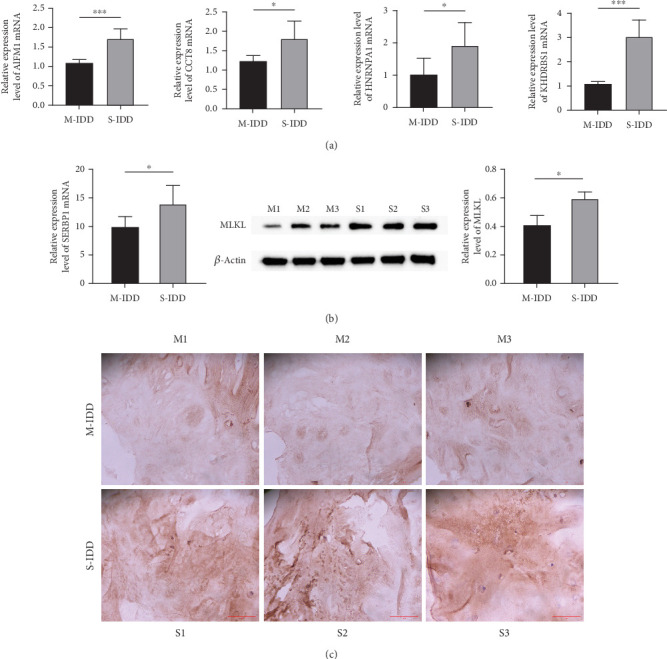
Patients with severe illness have higher levels of necrotic apoptosis. (a) qRT-PCR detection of key gene expression. (b) Western blot detection of MLKL activity (randomly select three sets of samples). (c) IHC detection of necrotic apoptosis levels in nucleus pulposus tissue (randomly select three sets of samples).

To validate our analysis, we collected lesion tissues from IDD patients and examined the expression levels of key genes. qRT-PCR results indicated that *AIFM1*, *CCT8*, *HNRNPA1*, *KHDRBS1*, and *SERBP1* were expressed at higher levels in S-IDD patients compared to M-IDD patients (Figure 5(a)). Additionally, we analyzed necrotic apoptosis levels, finding that p-MLKL levels in NP tissues were lower in M-IDD patients than in S-IDD patients (Figure 5(b)). IHC assays similarly showed higher MLKL expression in S-IDD patients (Figure 5(c)), suggesting increased necrotic apoptosis in severe cases. These findings imply that AIFM1, CCT8, HNRNPA1, KHDRBS1, and SERBP1 are crucial targets for predicting and treating IDD, potentially influencing its progression by regulating necrotic apoptosis in IVD.

## 4. Discussion

IDD plays a crucial role in spine-related diseases, and current treatments struggle to reverse its progression. Although numerous studies have explored the mechanisms of IDD, the underlying processes remain unclear. Increasing evidence suggests that necrotic apoptosis significantly contributes to IDD, potentially exacerbating its development by reducing the number of NP cells. The mechanisms linking necroptosis and IDD are still not fully understood, highlighting the importance of exploring potential necroptosis targets for early diagnosis and treatment of IDD.

In this study, we utilized IDD and normal tissue samples from GSE34095 and GSE124272 for differential gene screening, identifying 62 shared DEGs. By intersecting these with 643 NRGs, we identified five DE-NRGs: AIFM1, CCT8, HNRNPA1, KHDRBS1, and SERBP1. Further analysis revealed that these key genes effectively distinguish between IDD and normal samples, with AUC values exceeding 0.7. Additionally, using online databases miRWalk and starBase, we predicted the ceRNA relationships of these DE-NRGs and constructed both the ceRNA regulatory network and the regulatory network of the key genes. We also employed CLUE COMMAND to predict potential therapeutic drugs targeting these genes, aiming to improve IDD treatment in the future. Moreover, we verified the expression levels of the five DE-NRGs in vitro, finding higher expression in S-IDD compared to mild cases. This suggests that AIFM1, CCT8, HNRNPA1, KHDRBS1, and SERBP1 are crucial targets for IDD improvement, warranting further in-depth research.

The AIFM1 is a protein located in mitochondria and nuclei. It participates in caspase-independent apoptosis through nuclear fragmentation and promotes chromosome condensation and fragmentation [14]. In mitochondrial oxidative stress diseases and cancer, AIFM1 is linked to apoptosis and cancer progression [15–20]. AIFM1 has been reported to promote apoptosis in arthritis and IDD. For instance, silencing miR-766-3p, which promotes chondrocyte apoptosis in osteoarthritis, can be reversed by silencing AIFM1 [21]. Silencing AIF expression via siRNA significantly alleviated compression-induced programmed necrosis of myeloid cells [22]. Importantly, a recent study identified AIFM1 as a novel key downstream target of SIRT5. Under excessive mechanical stress, decreased SIRT5 expression leads to increased succinylation of AIFM1, impairing the activity of the AIFM1–CHCHD4 complex in NP cells and promoting IDD [23].

The cytoplasmic chaperonin-containing t-complex polypeptide 1 (CCT), also known as the t-complex polypeptide 1 (TCP1) ring complex (TRiC), consists of two identical stacked rings, each containing eight different proteins. CCT8 is a subunit of the CCT complex, containing gene sequences common to all other CCT subunits associated with ATPase activity [24]. Numerous studies have demonstrated that CCT8 plays a significant role in tumor progression in B-cell non-Hodgkin's lymphoma [25] and glioma [26]. CCT8 is also recognized as a potential biomarker for invasion and metastasis in colorectal, hepatocellular, and pancreatic cancers [27]. Beyond its involvement in tumor progression, CCT8 is essential for normal T cell biology. Loss of CCT8 function in T cells disrupts normal protein homeostasis and the dynamic formation of nuclear actin filaments, reducing the ability to manage activation-induced cellular stress. Additionally, T cells require CCT function to be polarized by Th2 to generate a protective immune response [28]. However, the role of CCT8 in IDD has not been reported.

HNRNPA1 is a widely expressed RNA-binding protein and the most abundant member of the hnRNP family. HNRNPA1 has been extensively studied in cancer, where it influences cell proliferation, invasiveness, metabolism, and stress adaptation by regulating the expression and translation of key genes associated with tumorigenesis and cancer progression [29–31]. In hepatocellular carcinoma (HCC), HNRNPA1 contributes to development by regulating cell cycle, mitochondrial dynamics, and necrotic apoptosis pathways [32]. Additionally, HNRNPA1 plays a role in counteracting the posttranscriptional regulation of cellular senescence. It mediates the overexpression of mitochondrial MFN1 and MFN2 in aged skeletal muscle, promoting an imbalance in mitochondrial fission and fusion, leading to morphological changes and accelerated senescence [33]. Our findings confirm that HNRNPA1 is significantly enriched in cell cycle, DNA replication, and nuclear DNA replication pathways, suggesting that HNRNPA1 may contribute to the development of IDD primarily by affecting the cell cycle.

KHDRBS1, also known as Sam68, is involved in mRNA splicing prior to signal transduction and is part of the signal transduction–related protein family. KHDRBS1 acts as an oncogenic splicing factor, linked to metabolic functions and diseases such as premature menopause and X-linked hereditary ataxia [34]. This aligns with the splicing bodies' pathway identified in our KEGG analyses. Selective splicing of KHDRBS1 can generate multiple transcript variants; improper regulation of this splicing increases susceptibility to primary ovarian insufficiency [35]. KHDRBS1 serves as a prognostic marker for kidney renal papillary cell carcinoma (KIRP), lung adenocarcinoma (LUAD), and acute myeloid leukemia (LAML) [34, 36, 37]. Abnormal expression of KHDRBS1 directly impacts cancer patient prognosis [38]. Importantly, Sam68 activates NF-*κ*B, promoting apoptosis of articular chondrocytes in arthritis patients, making it a potential target for treating joint-related diseases [39].

SERBP1 is a conserved RNA-binding protein involved in various cellular physiological processes, including cell division, DNA damage response, and cancer development [40]. It influences homologous recombination–mediated DNA repair by regulating the translation of S-phase CtBP-interacting proteins (CtIPs), leading to cell death [41]. SERBP1 also protects male germ cells from heat shock–induced apoptosis by scavenging arsenite and heat shock–induced stress granules (SGs) in mammalian cells [40]. Interestingly, overexpression of SERBP1 in the HEK293T cell line induces changes in genes related to cell proliferation, apoptosis, and the cell cycle [42]. Similarly, in HeLa cells, SERBP1 overexpression affects apoptosis levels by regulating the expression and selective splicing of genes involved in cellular and metabolic processes [43]. Our findings show significant upregulation of SERBP1, suggesting its potential involvement in the development of IDD through regulation of cellular processes.

Our current study has several limitations. First, all analyses were based on data mining using bioinformatics algorithms. Although we collected clinical samples for target validation, we could not assess the specific expression of key genes in IDD or establish a preventive and diagnostic standard due to the absence of a normal control group. Second, further investigation into the exact molecular mechanisms of candidate necrotic apoptosis-related genes in IDD is needed through in-depth in vitro and in vivo experiments. Lastly, the retrospective nature of this study limits its clinical value, highlighting the need for multicenter or prospective studies to elucidate the relationship between necrotic apoptosis and IDD.

## 5. Conclusion

In this study, NRGs *AIFM1*, *CCT8*, *HNRNPA1*, *KHDRBS1*, and *SERBP1* were suggested to be the key targets for IDD risk and drug therapy.

## Figures and Tables

**Table 1:** Primer sequence for qRT-PCR experiment.

**Genes**	**Forward primer**	**Reverse primer**
AIFM1	AAGTCAGACGAGAGGGGGTTA	GCCAACTCAACATTGGGCT
CCT8	AGGAGGGAGCGAAACACTTTT	GTTGCTGCATCGTTTGTCACA
HNRNPA1	TCAGAGTCTCCTAAAGAGCCC	ACCTTGTGTGGCCTTGCAT
KHDRBS1	GGAGCCAGAGAACAAGTACCT	CATGGCGTGAGTGAAGGAC
SERBP1	CCTGGGCACTTACAGGAAGG	GGTCCGATTCGTCGTCAAATAAC

**Table 2:** Expression of DE-NRGs.

**Symbol**	**logFC.GSE34095**	**P** **.Value.GSE34095**	**logFC.GSE124272**	**P** **.Value.GSE124272**
AIFM1	0.40296386	0.005614424	0.196797333	0.008034455
CCT8	0.448245424	0.025960368	0.634642	0.038177133
HNRNPA1	0.48114945	0.002150278	0.183479	0.030357721
KHDRBS1	0.35773406	0.046025829	0.275823333	0.03985657
SERBP1	0.66849788	0.043497053	0.274112333	0.002809306

## Data Availability

Data are available on request from the authors.

## Nomenclature

AML: acute myeloid leukemia
ANN: artificial neural network
DCA: decision curve analysis
DEGs: differentially expressed genes
DE-NRGs: differentially expressed necroptosis-related genes
GO: Gene Ontology
IDD: intervertebral disc degeneration
KEGG: Kyoto Encyclopedia of Genes and Genomes
KIRP: kidney renal papillary cell carcinoma
LR: logical regression
LUAD: lung adenocarcinoma
NP: nucleus pulposus
NRGs: necroptosis-related genes
OV: ovarian serous cystadenocarcinoma
RF: random forest
RIPK: receptor-interacting protein kinase
SNP: single nucleotide polymorphism
SVM: support vector machine
Tregs: T cell regulation
